# Predicting Incursion of Plant Invaders into Kruger National Park, South Africa: The Interplay of General Drivers and Species-Specific Factors

**DOI:** 10.1371/journal.pone.0028711

**Published:** 2011-12-14

**Authors:** Vojtěch Jarošík, Petr Pyšek, Llewellyn C. Foxcroft, David M. Richardson, Mathieu Rouget, Sandra MacFadyen

**Affiliations:** 1 Department of Ecology, Faculty of Sciences, Charles University in Prague, Prague, Czech Republic; 2 Department of Invasion Ecology, Institute of Botany, Academy of Sciences of the Czech Republic, Průhonice, Czech Republic; 3 Conservation Services, South African National Parks, Skukuza, South Africa; 4 Centre for Invasion Biology, Department of Botany and Zoology, Stellenbosch University, Stellenbosch, South Africa; 5 Department of Plant Science, University of Pretoria, Pretoria, South Africa; Michigan State University, United States of America

## Abstract

**Background:**

Overcoming boundaries is crucial for incursion of alien plant species and their successful naturalization and invasion within protected areas. Previous work showed that in Kruger National Park, South Africa, this process can be quantified and that factors determining the incursion of invasive species can be identified and predicted confidently. Here we explore the similarity between determinants of incursions identified by the general model based on a multispecies assemblage, and those identified by species-specific models. We analyzed the presence and absence of six invasive plant species in 1.0×1.5 km segments along the border of the park as a function of environmental characteristics from outside and inside the KNP boundary, using two data-mining techniques: classification trees and random forests.

**Principal Findings:**

The occurrence of *Ageratum houstonianum*, *Chromolaena odorata*, *Xanthium strumarium*, *Argemone ochroleuca*, *Opuntia stricta* and *Lantana camara* can be reliably predicted based on landscape characteristics identified by the general multispecies model, namely water runoff from surrounding watersheds and road density in a 10 km radius. The presence of main rivers and species-specific combinations of vegetation types are reliable predictors from inside the park.

**Conclusions:**

The predictors from the outside and inside of the park are complementary, and are approximately equally reliable for explaining the presence/absence of current invaders; those from the inside are, however, more reliable for predicting future invasions. Landscape characteristics determined as crucial predictors from outside the KNP serve as guidelines for management to enact proactive interventions to manipulate landscape features near the KNP to prevent further incursions. Predictors from the inside the KNP can be used reliably to identify high-risk areas to improve the cost-effectiveness of management, to locate invasive plants and target them for eradication.

## Introduction

Biological invasions impact all ecosystems [1]–[4] and although the type of habitat plays an important role in shaping invasion patterns in modern landscapes [5]–[12], very few habitats are free from alien plants [8], [13]. This also holds for protected areas at both regional [14] and global scales where the protection of biodiversity and ecosystem function is a fundamental goal. There is no up-to-date global synthesis of invasions in protected areas, but more than two decades ago an assessment showed that many nature reserves around the world harbored large numbers and densities of invasive species [15]. Although formal protection of ecosystems reduces some drivers of global environmental change, such as extensive transformation of land cover, many anthropogenic threats to biological diversity are not removed by establishing formal protected areas. Invasions by alien species are one such threat, and biological invasions are increasing in importance as threats to biodiversity in most protected areas. This is because human activities and land use in areas surrounding protected areas are key drivers of invasions within the protected areas, by providing sources of propagules of alien species and in other ways. Measures adopted to meet conservation goals such as establishing networks of protected areas and improving connectivity through the creation of corridors [16]–[17] do little to protect such areas from increasing threats from invasive species [18]–[20]. Indeed, some types of linkages may even exacerbate problems, e.g. river networks acting as conduits of plant invasion by supplying propagules and providing pathways for long-distance dispersal of alien species [21]–[22].

For protected areas with systematic management strategies for dealing with biological invasions, initiatives should generally focus on early detection and eradication, and focused action is usually only applied to the species that are likely to have greatest negative impacts on ecosystem functioning. Although notions of maintaining buffer zones around protected areas are often included and some work has addressed invasions at the interface between protected areas and human-dominated systems [23]–[25], penetration of alien species into protected areas, or what would constitute an effective and sustainable buffer to reduce incursions of alien plants only started to be addressed recently [26]. A generalized framework for synthesizing theories of ecological boundaries [27], suggests that three processes must considered: type of flow (e.g. organism movement through the landscape and thus across boundaries), patch contrasts (e.g. the difference in juxtaposed land use types), and boundary structure (the nature of the boundary which influences the movement of organisms). Therefore, in assessing the permeability of protected area boundaries to incursions by invasive species, we must consider factors reflecting both characteristics of the surrounding landscapes outside the park and those from within the protected area limits, adjacent to the park boundary.

Overcoming boundaries is crucial for incursion of an alien species and its successful naturalization and invasion within the protected area, a process that requires overcoming dispersal, reproductive and spread barriers [28]–[29]. There are surprisingly few studies of such incursions in the plant invasion literature. Two previous papers from widely separated geographical locations in Central Europe [23] and South Africa [26] have however shown that protected areas' boundaries act as an effective barrier against incursion of invasive species. Most invasive species reached protected areas from surrounding landscapes after the establishment of the protected area [23], and the rate of incursion and its determinants can be predicted based on landscape characteristics. This was shown for the Kruger National Park, South Africa, where the risk of incursion of invasive plants was accurately quantified. The density of invasive plants was found to decline rapidly beyond 1500 m inside the park, and the park boundary served to limit the spread of alien plant species. The degree of boundary permeability could be explained by a few characteristics of the landscape outside the park: water run-off, density of major roads, and the presence of natural vegetation. Of the metrics characterizing human impacts and disturbance, only the density of major roads outside the park played a significant role [26].

However, in searching for the role of generally valid drivers of invasions at various scales, studies rely on whole alien floras and faunas (e.g. [19], [30]–[33], or multispecies assemblages, and pay less attention to factors determining the success of individual species (but see [34]). This is because studies based on large data sets, in terms of species numbers, provide a more reliable basis for inferring generic patterns. Yet, it is important to investigate the extent to which results from multi-species studies apply to individual species, for which results can be interpreted in terms of autecology, habitat affinity, response to resources, species traits and other factors that are known to mediate invasiveness. Effective management interventions are often best formulated with particular species in mind [35].

The present paper therefore uses the general drivers of incursion of invasive plant species through the boundary of Kruger National Park that were identified in the previous paper [26] as a standard, and seeks to determine whether (and if so, then how) models for individual species deviate from this general pattern. The main aims are (i) to quantify, for individual species, the correspondence between determinants of incursion identified by the model based on the multispecies assemblage, and those identified by species-specific models; (ii) investigate whether the predictive power of models for those individual species that fit the multispecies general model can be improved by using additional factors; (iii) to assess for individual species the relative importance of predictors of incursions outside the park where landscape characteristics can be manipulated to some extent, and inside the park where this is not possible.

## Materials and Methods

### Study area

The study was carried out in Kruger National Park, South Africa (KNP), a large protected area that provides unique opportunities for gaining insights on incursions of invasive alien plants at a large spatial scale. The area is appropriate for such an exercise because of the unique detailed data that are available on alien plant species distribution [36] and features known to mediate plant invasions in and around the park [26]. Kruger National Park, located in the north-eastern region of South Africa, was founded in 1898 and covers an area of ∼20,000 km^2^. More than 370 non-native species have been recorded to date [37]. In response to the escalating importance of plant invasions, KNP has initiated a number of programs aimed at preventing and mitigating incursions of non-native species [38]–[39], and detailed data on the distribution of these species have been collected as part of long-term monitoring since 2004 [36]. The ecology of plant invasions has been intensively studied for more than a decade (e.g., [26], [34], [36], [40]–[41] and references cited in these papers).

Our study on the role of boundaries in filtering alien plant invasions focuses on the western and southern boundaries of KNP. The northern (Limpopo River) and eastern (border with Mozambique) boundaries were excluded from this analysis. This delimitation was based on the assumption that propagules of non-native species arrive mainly from the western side of the KNP because (i) all rivers flowing through the park flow from west to east [40], and (ii) tourism linkages, such as entrance gates, were developed primarily along the western and southern boundaries. Data from areas outside South Africa (Mozambique in the east and Zimbabwe in the north) does not match those from South Africa in terms of coverage and thoroughness. Also, the Limpopo River is an extensive drainage basin of which the KNP only has a minor portion (4%); including this edge would thus distort the effects explored in our study.

### Alien species data

Data on the occurrence of alien species and various other features are collected in KNP by approximately 120 field rangers during their daily patrols using a hand held personal computer (PDA) device, with customized software (CyberTracker; [26], [36], [42]–[43]. Records are taken randomly as rangers move through the field, stopping to record features of interest as they are encountered. Apart from the presence of alien plants, rangers also record animal sightings, water availability, carcasses, tracks, etc [36], [42]. We distinguished (i) presence points, which were records with the occurrence of a non-native plant indicated by a ranger, and (ii) absence points, where a record has been made but for a feature other than a non-native plant. This is based on assumption that had an alien plant been present at the same point as the other sightings, it would have been recorded by the ranger [36]. This assumption is justified, because the data set included the most abundant and conspicuous alien species that are reliably recognized by trained rangers: *Opuntia stricta*, *Lantana camara*, *Chromolaena odorata* and *Parthenium hysterophorus* [36]. These species together account for 82% of all alien plant records in KNP and represent thus a highly representative sample.

The large spatially-explicit dataset gathered by the rangers covers the entire KNP [36] and includes >27,000 presence points and >2 million absence points. For our analyses we divided the western and southern park boundary into 1-km-wide segments perpendicular to the boundary, running towards the park interior to a distance of 1.5 km (hereafter referred to as segments, each of 1.0×1.5 km in size); in total, 637 boundary segments were created (see [26]: their Fig. 1). The occurrence (presence or absence) of individual alien species in these segments was used for further evaluations, separately for the following six species with sufficient numbers of presences to allow for statistical analysis: *Ageratum houstonianum*, *Chromolaena odorata*, *Xanthium strumarium*, *Argemone ochroleuca*, *Opuntia stricta* and *Lantana camara* (Table S1).

**Figure 1:**
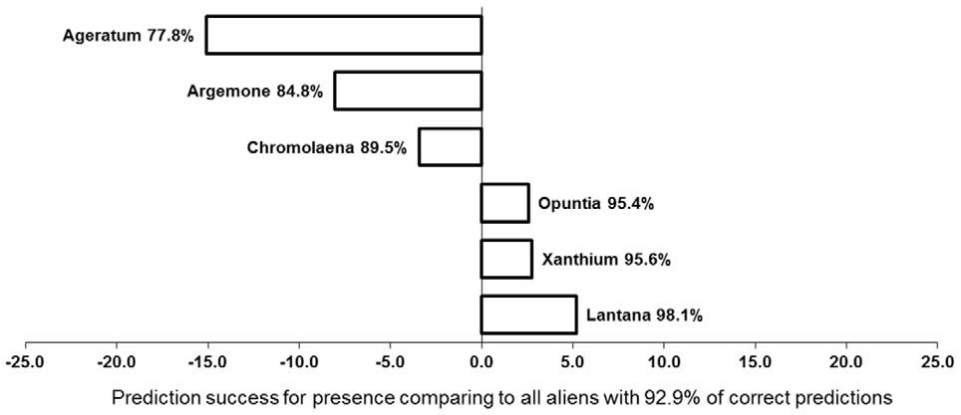
Prediction success for presences (%) of the individual species. *Ageratum houstonianum*, *Argemone ochroleuca*, *Chromolaena odorata*, *Opuntia stricta*, *Xanthium strumarium* and *Lantana camara* are evaluated based on scoring, i.e. dropping the data separately for each of the six species down the previously established optimal multi-species tree (see Foxcroft et al. 2011, their Fig. 3), further termed “the general model”. In the general model, probability of presence was determined by mean annual runoff from the surrounding watershed and density of major roads within a 10 km radius outside the KNP boundary. Prediction success for all species presences, describing percentage of successful predictions, was 92.9% (vertical line at zero point of x-axis). Sensitivity, describing proportional ability of the general model to predict that the species is present when the actual dataset applied to new data, was 0.92. Optimal models for the individual species use the same building rules, the same segments for presence and absence of the species and the same environmental characteristics as the general model.

### Environmental data

To explain the incursions of alien plants into KNP, we used explanatory variables characterizing environmental conditions inside and outside the park. The variables represent either environmental (e.g. water run-off) or anthropogenic factors (e.g. roads); most of them assumed to be surrogates of propagule pressure. The variables used inside the park were expressed for the 1×1.5 km segments along the boundary (see [26], their Fig. 1). Environmental conditions outside the park were summarized for sections starting opposite to the boundary segments and running into the landscape surrounding the park, and were expressed for 1, 5, 10 and 50 km radius outside the park boundary.

The variables outside KNP included those related to (i) Traffic: density of major roads (defined as the main tourist tar roads) and of all roads within 1, 5, 10, 50 km of boundary [km/km^2^]; (ii) Land use: % of natural areas (untransformed landscapes, although probably grazed by livestock), cultivated areas (agricultural land), urban areas (including towns and informal/rural settlements) and degraded areas (transformed by erosion, i.e. gullies and bare soil, loss of plant cover and other disturbances) in 1, 5, 10 and 50 km radius from the boundary, and % of plantations (commercial plantation forests) in 10 and 50 km radius. (iii) Presence of protected areas adjacent KNP; (iv) Run-off from quaternary watershed [43], given only for those segments for which a main river (Limpopo, Luvuvhu, Shingwedzi, Letaba, Olifants, Sabie, Crocodile River) intersected it and the measures included: mean annual runoff [million m^3^/quaternary watershed/annum], and river runoff category [none, low, medium, high]. (v) Vegetation productivity expressed as NDVI (Normalized Difference Vegetation Index) mean value, which is a measure of the amount of green vegetation i.e. photosynthetically active material, and is used as a proxy for above-ground net primary production.

The variables inside KNP included (i) presence of major roads, all roads, camps and gates; (ii) presence of main river and all rivers; and (iii) vegetation type, expressed as landscape units which are defined as areas with a specific geomorphology, macroclimate, soil and vegetation pattern, and associated fauna [44], [45]. The following landscape units were present in the segments analysed: Lowveld Sour Bushveld of Pretoriuskop (unit ID = 1); Malelane Mountain Bushveld (2); Thickets of the Sabie & Crocodile Rivers (4); Mixed *Combretum*/*Terminalia sericea* woodland (5); *Combretum*/*Colophospermum mopane* woodland of Timbavati (6); Olifants River Rugged Veld (7); Phalaborwa Sandveld (8); *Colophospermum mopane* woodland/savanna on basic soil (9); Letaba River Rugged Veld (10); Tsende Sandveld (11); *Colophospermum mopane*/*Acacia nigrescens* savanna (12); *Acacia welwitschii* thickets on Karoo sediments (13); Punda Maria Sandveld on Cave Sandstone (16); *Sclerocarya birrea* subsp. *caffra*/*Acacia nigrescens* savanna (17); Thornveld on gabbro (19); *Colophospermum mopane* shrubveld on gabbro (24); *Adansonia digitata*/*Colophospermum mopane* Rugged Veld (25); *Colophospermum mopane* shrubveld on calcrete (26); Limpopo/Luvuvhu Floodplains (28); Lebombo South (29); *Pterocarpus rotundifolius*/*Combretum collinum* woodland (33); Punda Maria Sandveld on Waterberg sandstone (34).

### Statistical analysis

#### Response and predictor variables

To ensure the comparability of results yielded by the multispecies model based on all species from a previous study (further referred to as “the general model”) with the individual species models addressed here, we used exactly the same data set as in [26]. The presence and absence of alien species in the 637 contiguous, 1 km wide segments was used as the response variable and 36 environmental characteristics measured within and outside KNP were included as predictor variables.

#### Predictive mining

To analyze the presence and absence of the alien species studied in the segments as a function of the environmental characteristics, we applied classification and regression trees [46]–[48] and random forests [49]–[50] using CART® v.6.0 and Random Forests® v. 2 in the statistical software Salford Predictive Mining Suite. In these methods, data are successively split along coordinate axes of the predictors, represented by the environmental characteristics, so that at any node the split that maximally distinguishes the response variable is selected (presence or absence per segment), in the left and the right branches. This was done using binary recursive partitioning, with a best split made based on default Gini impurity measure [51]–[52].

The data-mining techniques enable one to make predictions from the data and to identify the most important predictors by screening a large number of candidate variables, without requiring any assumptions about the form of the relationships between predictors and the response variable, and without *a priori* formulated hypotheses [53]. These methods are also more flexible than traditional statistical analyses because they can reveal more than only linear structures in the dataset, and can resolve complex interactions. Importantly, these techniques are nonparametric and thus not affected by spatial autocorrelations and by collinearity of the predictor variables [52], [54]. The ranking of predictors' variable importance thus guards against the elimination of variables which are good predictors of the response, and may be ecologically important, but are correlated with other predictors.

#### Classification trees

Classification trees provide intuitive insight into the kinds of interactions between the predictors. They are represented graphically, with the root standing for undivided data at the top, and the terminal nodes, describing the most homogeneous groups of data, at the bottom of the hierarchy. The quality of each split was expressed by its improvement value, corresponding to the overall misclassification rate at each node, with high scores of improvement values corresponding to splits of high quality. Surrogates of each split, describing splitting rules that closely mimicked the action of the primary split, were assessed and ranked according to their association values, with the highest possible value 1.0 corresponding to the surrogate producing exactly the same split as the primary split. Because high categorical predictors have higher splitting power than continuous predictors, to prevent the high categorical predictor type of dominant vegetation inside the park (22 categories) to have inherent advantage over continuous variables, penalization rules for high category variables [51] were applied.

Making a decision on when a tree is complete was achieved by growing the largest tree and then examining smaller trees obtained by gradually decreasing the size of the maximal tree [46]. A single optimal tree was then determined by testing for misclassification error rates for the largest tree and for every smaller tree. Cross-validation was used to obtain estimates of relative errors of these trees. These estimates were then plotted against tree size, and the optimal tree chosen both based on the minimum cost tree rule, which minimizes the cross validated error (the default setting in CART v 6.0; [51], and based on the one-SE rule, which minimizes cross-validated error within one standard error of the minimum [46]. A series of 50 cross-validations were run, and the modal (most likely) single optimal tree chosen for description [55].

#### Species selection and cross-validation procedure

Because our data set comprised 637 records for each species (presence/absence in the individual segment), with fewer records for presence than absence, it was too small for reliable testing by the use of a learning (i.e. training) and a test sample. Consequently, for reliable testing of optimal trees only cross validation could be used [48]. Cross-validation involves splitting the data into a number of smaller samples with similar distributions of the response variable. Trees are then generated, excluding the data from each subsample in turn. For each tree, the error rate is estimated from the subsample excluded in generating it and the cross-validated error for the overall tree is then calculated.

The use of cross-validation restricted the number of tested species because cross-validation results become less reliable when the number of cross-validated folds is reduced below 10 [46], and because balanced classes should be used for each cross-validation fold with the rare records ([48], p. 93). We therefore included only those invasive alien species with 18 or more recorded presences in the segments in our analyses, which enabled to use 9-fold cross-validation with at least two presence records in each fold for each species: *Ageratum houstonianum* (18 presences in segments), *Chromolaena odorata* (19), *Xanthium strumarium* (23), *Argemone ochroleuca* (33), *Opuntia stricta* (88) and *Lantana camara* (156).

#### Scoring and species-specific classification trees

For the six most abundant invasive alien species chosen, we tested the predictive power of the previously established general model for all alien species treated together [26]. In this general model, the default minimum size of the splitting node was 10 cases, and the optimal tree was determined based on 10-fold cross-validation. The model was determined for records in the same segments and with probability of occurrence assessed using the same environmental characteristics as in this study. In this previously established model, the mean annual water runoff >6 million m^3^/annum from the watershed outside the park explained the greatest proportion of variance in alien records. Segments with less than 6 million m^3^/annum runoff were more likely to have alien species present only in areas with >0.1 km/km^2^ major road density within 10 km outside the park boundary (Fig. 1).

The testing of predictive power of this previously established general model was done by scoring, i.e. by dropping the data separately for each of the six invasive species addressed in this study from the previously established optimal tree. Each observation was processed case by case, beginning at the root node. The splitting criteria for the general optimal tree were applied, and in response to each yes/no question, the case for each species moved left or right down the tree until it reached the terminal node.

We then used the binary classification trees separately for each of the six species, applying exactly the same procedures as for the general model, except that 9-fold instead of 10-fold cross-validations were used. These analyses aimed to show to what extent species-specific classification trees are able to improve predictions yielded by the general model.

#### Measures of predictions

Because, unlike in the general model with 253 presences and 384 absences in the segments, for the individual species the presence/absence classes were highly unbalanced (i.e. very few presences records), all analyses were conducted with balanced class weights [48], assuring that presence and absence classes were treated as equally important for the purpose of achieving classification accuracy. All the data for individual species could then be evaluated based on comparisons of species presences. We evaluated the misclassification rate [55] and prediction success, expressed as 100 – percent of misclassification rate, for presences of the individual species in the segments. These values were expressed based on learning samples, i.e. the samples not used to build the trees for assessment of cross-validation errors [55]. Following [56], we also evaluated sensitivity, i.e. the ability of the models to predict that the species is present when it is. The values of sensitivity were based on cross-validated samples, i.e. the best estimates of the misclassification that would occur if the classification tree were to be applied to new data, assuming that the new data were drawn from the same distribution as the learning data [51].

For the general classification model with relatively balanced presence/absence classes, we also evaluated variable importance based on improvement values at each split. The values were summed over each node and totaled, and scaled relative to the best performing variable. The variable with the highest sum of improvements was scored 100, and all other variables had lower scores ranking downwards towards zero. The scoring was done both based on standard variable importance ranking, i.e. including effects of surrogates, and using ranking based only on the primary splitters. In the standard ranking, a predictor variable can be considered highly important even if it never appears as a primary splitter because the method keeps track of surrogate splits in the tree growing process, and the contribution a variable can make in prediction is thus not determined only by primary splits. Comparing the standard variable importance rankings with considering only primary splitters thus can be very informative because variables that appear to be important but rarely split nodes are probably highly correlated with the primary splitters and contain very similar information [48].

#### Random forests and classification trees based on random forests ranking

The standard importance score of classification tree measures a variable's ability to mimic the chosen tree, but says nothing about the value of any variable in the construction of other trees. Thus, the rankings are strictly relative to a particular tree and changing that tree by removing a variable can result in substantial reshuffling of the rankings [51]. The ranking can be also quite sensitive to random fluctuation in the data [46]. To obtain a more reliable ranking of the variable importance values than is possible in the classification trees, we applied random forests [50], [52]. As in the case of classification trees, random forests were first applied for all invasive species treated together, and the predictive power of this general model was then tested separately for each species by scoring.

Random forests can be seen as an extension of classification trees by fitting many sub-trees to parts of the dataset and then combining the predictions from all trees. They are fitted on boot-strapped subsamples of the entire dataset, and observations that did not occur in a particular sample are left as out-of-bag observations. At a root node, a random sample of six predictors (equal to a square root of the number of predictors; [50]) was selected. At each subsequent node, another small random sample of six predictors was chosen, and the best split made. The tree continued to be grown in this fashion until it reached the largest possible size and then was used to predict the out-of-bag observations. The whole process, starting with a new bootstrap sample, was repeated 500 times, with all observations having equal probability of entering each bootstrap sample. The predicted presence/absence class for each observation was then calculated by majority vote of the out-of-bag predictions for that observation from the 500 simulated trees, with ties split randomly.

To assess the importance of the individual predictors in random trees, scaled relative to the best performing variable as in the classification trees, a novel out-of-bag method for determining variable importance, having very high classification accuracy, was applied. In this method, the values of each explanatory variable were randomly permuted for the out-of-bag observations, and the modified out-of-bag data were passed down the tree to get new predictions. The difference between the misclassification rate for the modified and original out-of-bag data, divided by the standard error, was a measure of the importance of the variable [50], [52]. The importance ranking of the individual predictors based on random forest was then used for predicting probability of presences of the individual species in alternative classification trees, examining the role of crucial factors from inside the KNP.

## Results

### Role of landscape structures outside KNP

Predicting presences of the six individual species (Fig. 1) by dropping the data for each species separately down the previously built optimal general tree based on all species ([26], their Fig. 3) yielded worse results than those based on the optimal general model for *Ageratum*, *Argemone* and *Chromolaena* analyzed separately, and better results for *Opuntia*, *Xanthium* and *Lantana*. Overall, the prediction success for presences yielded by the optimal general tree was very high, equal to 92.9% from the actual dataset, and similarly its sensitivity was also high , describing the proportional ability of the general model to predict that the species is present when the actual dataset is applied to new data, reaching the value of 0.92 (Fig. 1). The prediction success for the individual species ranged from 77.8% for *A*g*eratum* to 98.1% for *Lantana* (Fig. 1). All individual species were thus reliably predicted by the linear environmental landscape elements outside the KNP, both natural (rivers) and artificial (roads), that were identified by the optimal general tree built for all invasive species [26].

**Figure 2:**
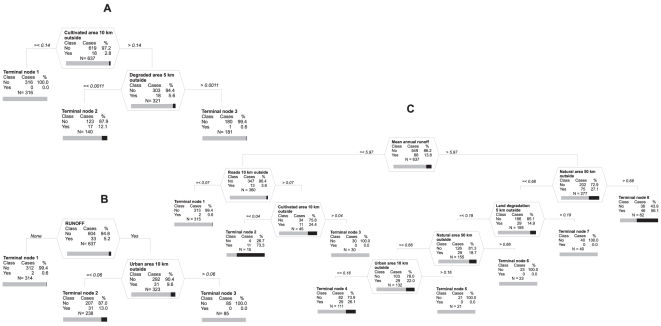
Single-species optimum classification trees for *Ageratum houstonianum* (A), *Argemone ochroleuca* (B) and *Opuntia stricta* (C). %, percentage of cases for each class; bars, representation of percentage of absent (grey) and present (black). Except for the root node (undivided data) at the top, the splitting variable name and split criterion is given above each node. Vertical depth of each node is proportional to its improvement value. (A) Prediction success for species presence 94.4%, sensitivity 0.78. (B) Prediction success 93.9%, sensitivity 0.82; the categorical splitter Water run-off can be equally well expressed by continuous splitter Water run-off (as in optimal multi-species tree), or a binary splitter from the inside of KNP Main river present/absent - both these surrogates have association value equal one and the same improvement value as the primary splitter RUNOFF. (C) Prediction success 97.7%, sensitivity 0.81.

When the same procedure as for building the optimal general tree was used for single-species optimal classification trees, but not limited to the set of predictors defined by the general model, prediction success for presences substantially increased by 16.6% and 9.1% for *Ageratum* and *Argemone*, respectively. As in the general tree, the presences of *Ageratum* and *Argemone* were best predicted by environmental factors outside KNP. However, *Ageratum* was not best predicted by linear landscape components, i.e. rivers and roads, and *Argemone* only partially. Single-species optimal tree for *Ageratum* indicated that this species is supported by the presence of cultivated land in larger distances from the KNP boundary and that of degraded areas close to it (Fig. 2A). The presence of *Argemone* was supported by high water runoff as for all the remaining species, but instead of density of major roads within 10 km radius outside KNP, it was supported by low urbanization within this radius (Fig. 2B). Except for *Opuntia*, the prediction of the remaining species (that were all already well predicted by the optimal general tree) was not improved by species-specific optimal trees. A small improvement of 2.7% for *O. stricta*, compared to the optimal general tree, was attributed to fine-tuning splits below the main splitters common for all species (Fig. 2C). In segments with low water runoff in the park, the presence of *Opuntia* was supported by a high density of all roads within 10 km radius outside the KNP boundary and by a low proportion of cultivated landscape within this radius; in segments with such properties, the incidence of *Opuntia* reached the highest value, being present in 73% of segments (terminal node 2). In segments with a high water runoff, *Opuntia* was present in as many as 56% of them, if they were surrounded by more than 68% of natural areas within 50 km radius outside the boundary (terminal node 5). In segments with a lower proportion of natural vegetation in the surrounding area, *Opuntia* was much less often present, and supported by a low level of land degradation in a 5 km radius outside the boundary and low urbanization (Fig. 2C).

Overall, the landscape features outside KNP – water runoff from surrounding watershed and road density within the 10 km radius, and to some extent also cultivated, degraded, urban and natural areas adjacent to the park – reliably predicted the presence of species in segments, and also enabled reliable predictions for new data.

### Role of main rivers and vegetation types inside KNP

**Figure 3:**
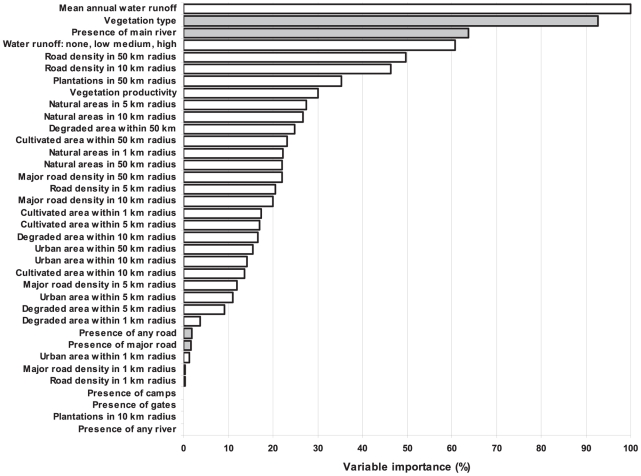
Ranking of importance values (%) for all invasive species. Ranking is scaled relative to the best performing variable based on out-of-bag method of random forests. White bars are predictors from the outside of Kruger National Park and grey bars from the inside.

Considering only primary splitters, the ranking of importance values of the optimal tree for all invasive species scored the mean water run-off from the watershed surrounding KNP 100%, and major road density in a 10 km radius outside the park 16%. However, a ranking which takes into account surrogates of primary splitters scored the dominant type of vegetation inside the park as the most important variable, suggesting that the vegetation type is strongly correlated with the primary splitters from the outside of the park. Indeed, ranking of the variable importance values based on random forests (Fig. 3) scored the vegetation type inside the park as the second most important predictor. This was followed by another variable from inside the park, the presence of a main river, which was the closest surrogate of the most important predictor, mean annual water runoff from the surrounding watershed. Moreover, the random forests built for all species perfectly matched random forests for the individual species, as revealed by 100% prediction success for presences of the individual species when dropping them individually down the random forests built for all species. The only exception was *Opuntia* for which this scoring recorded one misclassification case. The results thus show that instead of predicting the probability of presences of the individual species based on predictors from outside the park, an alternative prediction can be done using two predictors from inside the park: dominant vegetation type and the presence of a main river.

The optimal classification trees for the probability of presences of the individual species, built by using dominant vegetation types and presence of main rivers inside KNP, i.e. based on the two best predictors chosen by random forests, had on average by 9.3% higher sensitivity and only by 2.8% worse prediction success than for optimal trees built using all predictors, i.e. without their pre-selection by random forests. Relying on predictors describing landscape structures outside KNP, which were chosen by optimal trees from all 36 environmental variables, thus appeared approximately equally reliable as pre-selection of the two predictors from inside KNP by the random forest. However, the approach based on pre-selection of predictors by random forests appeared more reliable for predicting potential future invasions.

### Predictions of individual species based on pre-selected predictors from inside KNP

Using optimal trees based on pre-selection of the two most important predictors inside KNP, *Ageratum* occurred in all cases in segments with a main river (prediction success 100%) and should always occur in these segments when this prediction is also applied to a new data set (sensitivity 1). Alternatively to this prediction, *Ageratum* also occurred with 100% prediction success in seven vegetation types: Melale Mountain Bushveld, Thickets of the Sabie & Crocodile Rivers, Mixed *Combretum*/*Terminalia sericea* woodland, *Acacia welwitschii* thickets on Karoo sediments, *Sclerocarya birrea* subspecies *caffra*/*Acacia nigrescens* savanna, *Adansonia digitata*/*Colophospermum mopane* Rugged Veld, and Punda Maria Sandveld on Waterberg sandstone. However, this alternative prediction appeared less reliable when applied to new data (sensitivity 0.83).

Similarly, *Xanthium* was predicted reliably both by the presence of main rivers (prediction success 95.6% corresponding to one misclassification case; sensitivity 0.95) and by the vegetation types Lowveld Sour Bushveld of Pretoriuskop, Letaba River Rugged Veld, Tsende Sandveld, *Acacia welwitschii* thickets on Karoo sediments, Malelane Mountain Bushveld, *Pterocarpus rotundifolius*/*Combretum collinum* woodland, Thickets of the Sabie & Crocodile Rivers, Mixed *Combretum*/*Terminalia sericea* woodland, Olifants River Rugged Veld and Phalaborwa Sandveld (prediction success 100%). However, as for *Ageratum*, the vegetation types were less reliable when predicting future invasions (sensitivity 0.74). *Chromolaena* was predicted reliably by the presence of main river (prediction success 94.7% corresponding to one misclassification case; sensitivity 0.94,), but its prediction appeared unreliable (no optimal tree built) using the predominant vegetation types.

**Figure 4:**
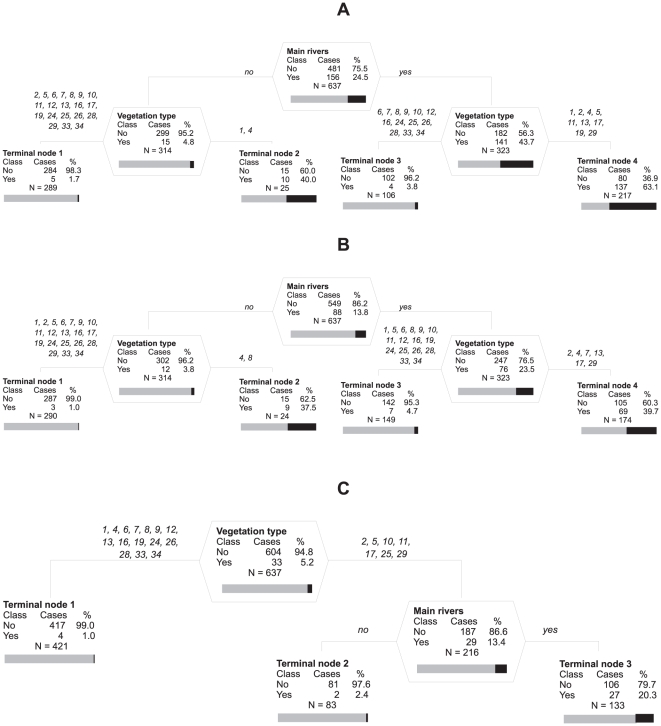
Optimal regression trees based on pre-selection of two most important predictors. The pre-selection of the two most important predictors, the dominant vegetation type and the presence of main river from inside KNP, is based on random forests. (A) *Lantana camara*, (B) *Opuntia stricta*, (C) *Argemone ochroleuca*. Identification numbers of vegetation types are: 1 Lowveld Sour Bushveld of Pretoriuskop, 2 Malelane Mountain Bushveld, 4 Thickets of the Sabie & Crocodile Rivers, 5 Mixed *Combretum*/*Terminalia sericea* woodland, 6 *Combretum*/*Colophospermum mopane* woodland of Timbavati, 7 Olifants River Rugged Veld, 8 Phalaborwa Sandveld, 9 *Colophospermum mopane* woodland/savanna on basic soil, 10 Letaba River Rugged Veld, 11 Tsende Sandveld, 12 *Colophospermum mopane*/*Acacia nigrescens* savanna, 13 *Acacia welwitschii* thickets on Karoo sediments, 16 Punda Maria Sandveld on Cave Sandstone, 17 *Sclerocarya birrea* subspecies caffra/*Acacia nigrescens* savanna, 19 Thornveld on gabbro, 24 *Colophospermum mopane* shrubveld on gabbro, 25 *Adansonia digitata*/*Colophospermum mopane* Rugged Veld, 26 *Colophospermum mopane* shrubveld on calcrete, 28 Limpopo/Luvuvhu Floodplains, 29 Lebombo South, 33 *Pterocarpus rotundifolius*/*Combretum collinum* woodland, 34 Punda Maria Sandveld on Waterberg sandstone. Values of prediction success and sensitivity are given in the text. Otherwise as in Fig. 2.

*Lantana* was reliably predicted (prediction success 94.2%; sensitivity 0.92) by splitting the prediction first based on occurrence of the main river, and then following vegetation types shown in Fig. 4A. This species occurred in as many as 63% of the segments with suitable vegetation types and the river present, but even if there was no river, presence of vegetation types suitable for invasion resulted in 40% probability of occurrence.

The model for *Opuntia* (prediction success 88.6%, sensitivity 0.89) had the same structure as that for *Lantana* and the negative effect of main river's absence could be compensated by the occurrence of a vegetation type suitable for invasion, as indicated by similar probability of this species presence, 37.5% and 39.7%, in terminal nodes without and with a main river, respectively (Fig. 4B).

*A*r*gemone* (prediction success 81.8%, sensitivity 0.79) was virtually absent from some vegetation types, while in some others it occurred with 20.3% probability provided that a main river flows through segments with these vegetation types (Fig. 4C).

### Predictions of species absences

**Figure 5:**
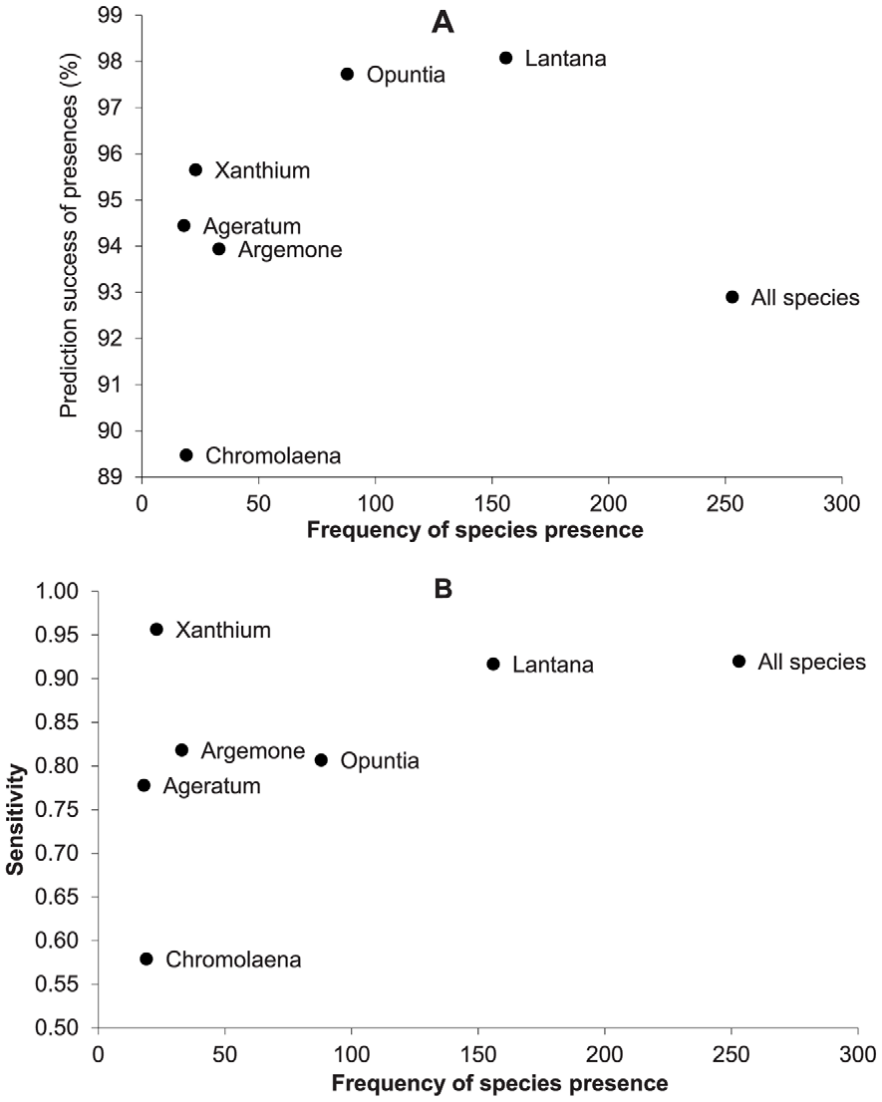
Prediction success (A) and sensitivity (B) of species presences in individual segments. The segments are from around the western and southern boundary of Kruger National Park and are calculated from general (based on all species) and species-specific optimal classification trees, plotted against actual number of species presences in the segments. Plots show that prediction success (A) and sensitivity (B) are independent of the species frequency: (A) Spearman's rank correlation r_s_ = 0.25; z = 0.57; P = 0.57; (B) r_s_ = 0.57; z = 1.36; P = 0.17.

**Figure 6:**
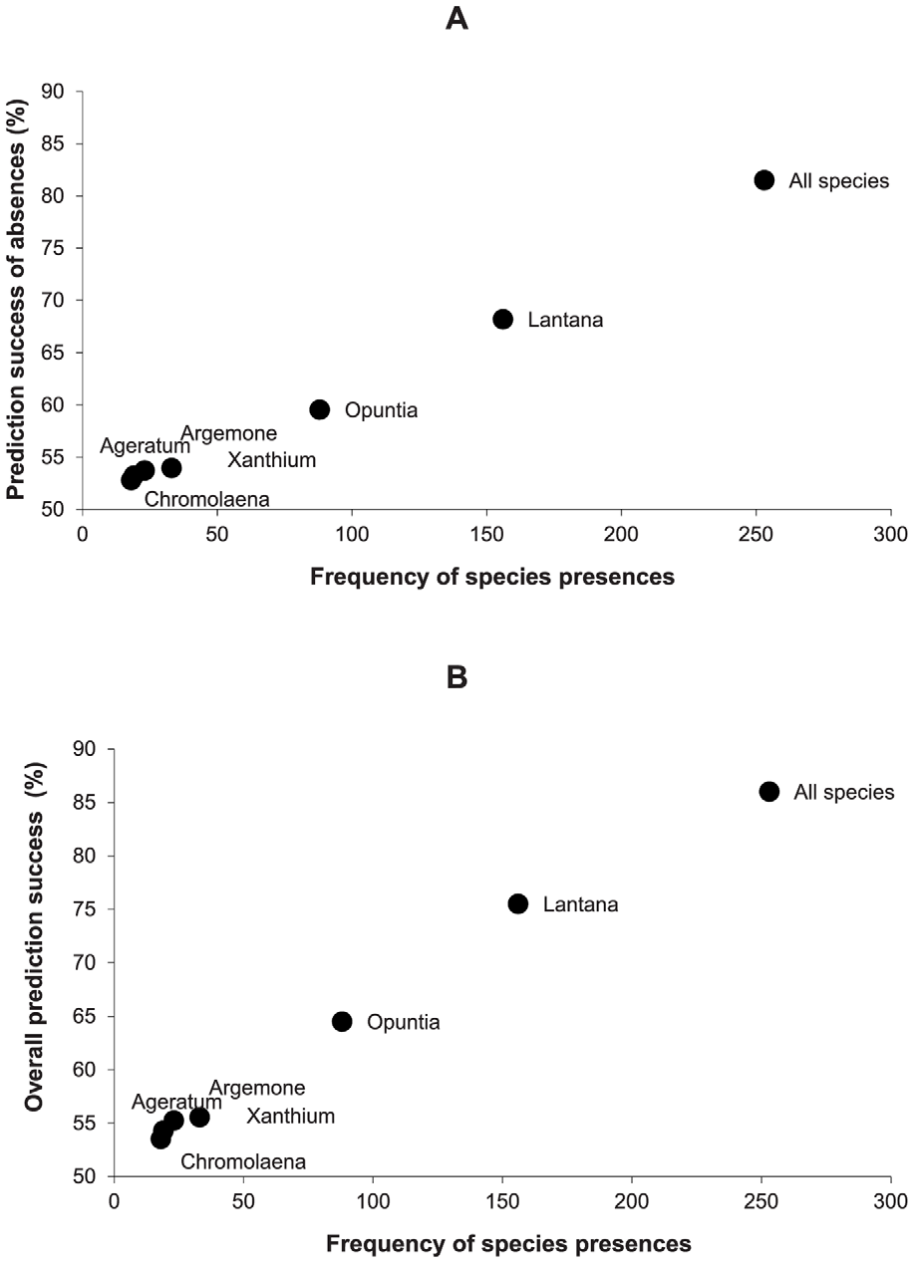
Prediction success of absences (A) and overall prediction success for presences and absences (B). Data presented as in Fig. 5. Both plots show that these predictions are strongly dependent on species frequency: Spearman's rank correlations for (A) and (B): r_s_ = 1; z = 2.41; P = 0.02.

Measures of species presences were independent of species frequencies in the individual segments (Fig. 5), and species presences were therefore reliably predicted even for infrequent species. However, it was not true for prediction success of species absences, and consequently, also not for the overall prediction success of presences and absences (Fig. 6). Thus, due to the increasing uncertainty of species predictions with decreasing species frequency, the true knowledge of segments which are unsuitable for the presence of the individual species remains largely unknown.

## Discussion

### How informative is the general model for predictions of individual species?

A previous study showed that for a large protected area, exemplified by South Africa's Kruger National Park, the risk of incursion of invasive plants can be successfully quantified and predicted to a high degree [26]. Overall, the general model established by using a multi-species data set in that study worked well for predicting the occurrence of the individual species analyzed in the present study. The results thus show that using a general model for predicting the likelihood of invasion by individual species seems to be generally useful, and can be applied also to other conservation areas. As boundaries are becoming increasingly important for buffering human impacts in protected area, further surveys and surveillance is likely to increase in these areas. Also, as gathering detailed data through monitoring is difficult and expensive, even collecting simple GPS localities of species can provide data on which powerful analyses can be done. These analyses can serve as a basis for important management recommendations, such as manipulating factors that determine the invasions of particular species or describing focal points for control of specific species.

It could be argued that the good fit between the models for individual species and the general one was partly due to the small number of species used to build the general model. Nevertheless, the general model was based on 26% of all alien species records in the park and included all the problem species in KNP, which makes it highly representative of incursion of alien species into KNP. Moreover, the ability of the previously established general multispecies model [26] to predict the occurrence of individual species did not depend on whether or not the given species was part of the original model. Although *Opuntia* and *Lantana* (both used to build the general model), performed better in terms of prediction than did *Argemone* and *Ageratum* (not used for the general model), the occurrence of *Chromolaena odorata*, which was included in the general model, was more poorly predicted than average, while that of *Xanthium* which not included, performed better. Testing the general model's validity for species which are not yet invasive but that may invade KNP in the future therefore seems plausible.

It is useful to evaluate the results for two groups of species separately, to obtain better insights into the value of our predictive models. (i) For those invasive species that already have a high number of records (*Opuntia*, *Lantana*) the high correspondence with the general model is not surprising because data on those species dominated the contribution to the general model. Yet, our current analysis shows that predictions for even such species can be further improved (although this applied only for *Opuntia* and the improvement was very small) by employing information on additional landscape features outside the park. (ii) For three species (*Ageratum*, *Argemone*, *Xanthium*), testing the performance of the general model was completely independent as these species were not involved in its construction. Nevertheless, even for the two less well performing species of this group (*Ageratum*, *Argemone*), the models using the same structure worked reasonably well, but could be substantially improved by employing landscape features outside the park.

When using predictors from inside the park, pre-selected by the random forest analysis, habitat type played an important role for all the species. We suggest that this is because habitats and associated vegetation types are important determinants of the success of establishment and invasion of species [13]. The affinity to habitat types is species-specific, can change following introduction to new environment [57], and reflects population processes, ecological requirements of the species and competitive interactions with species forming recipient communities. The response of the invading species to habitat structure and mosaic of vegetation types present in the target landscape therefore fine-tunes the effect of general drivers recruiting from mostly human-induced disturbances that create pathways and generate propagule pressure [58].

### Incursions of alien species into KNP are an ongoing process

Presences of even infrequent species can be predicted with reasonably high certainty but attempts to predict unsuitable habitats appear unreliable because uncertainty of the prediction of absences increases with species rarity. This can be attributed to the fact that alien species are still spreading across the park boundary and not all suitable segments are thus occupied (cf. [59]–[60]). Consequently, the more abundant the species, the more it saturates individual segments in which it occurs, making the assessment of its absence in segments more reliable. Also, in the general multispecies model with absences and presences nearly balanced in the segments, the prediction success of species presences was more than twice as good (misclassification rate 7.1%) as that for absences (18.5%). This suggests that the segments are saturated neither by individual invading species penetrating into KNP nor in terms of the entire alien flora, which makes future invasions of more alien species very likely.

### Past and future invasions: manipulate the former, watch the latter

From the above it follows that the model based on several of the most abundant invasive species in KNP [26] is generally sufficiently robust to be used for individual species with reasonable precision. This suggests that despite the differences in species traits and particular features of invasion dynamics that are unique to certain species, the major drivers of invasion act in a similar way and with comparable efficiency for most of the invasive species. Yet, individual species deviate from the general pattern to different degrees. Using information on vegetation types invaded can improve not only the prediction of the overall species occurrence but also paves the way for more precise prediction of future invasions. While the predictions based on factors from the outside and inside of KNP are complementary, and are approximately equally reliable for the prediction of current invasions, those from the inside are more reliable for predicting future invasions.

The specific information conveyed by each of the two sets of predictors could prove useful for management. Factors describing landscape structures outside KNP provide the basis for managing the surrounding countryside to minimize future invasions (see discussion in [26]), while inside-park predictions based on main rivers and dominant vegetation types can be used to prioritize localities and target them for more intensive monitoring, rapid-response efforts for emerging invaders, and other management actions for well-established alien species. This has potentially important economic consequences – by focusing only on a subset of vegetation types identified as high-risk for invasion along the park boundary, and fine-tuning the target areas by using information on the presence of rivers, management can be made more cost effective. Combining complementary predictors from the outside and inside of a conservation area thus appears a promising general management strategy.

## Supporting Information

Table S1 Characteristics for the six focal species used in this study.
(DOCX)

## References

[1] Millennium Ecosystem Assessment (2005). Ecosystems and human well-being.

[2] Vilà M, Basnou C, Pyšek P, Josefsson M, Genovesi P (2010). How well do we understand the impacts of alien species on ecosystem services? A pan-European, cross-taxa assessment.. Front Ecol Environ.

[3] Vilà M, Espinar JL, Hejda M, Hulme PE, Jarošík V (2011). Ecological impacts of invasive alien plants: A meta-analysis of their effects on species, communities and ecosystems.. Ecol Lett.

[4] Pyšek P, Richardson DM (2010). Invasive species, environmental change and management, and health.. Ann Rev Environ Res.

[5] Stohlgren TJ, Binkley D, Chong GW, Kalkhan MA, Schell LD (1999). Exotic plant species invade hot spots of native plant diversity.. Ecol Monogr.

[6] Stohlgren T, Jarnevich C, Chong GW, Evangelista PH (2006). Scale and plant invasions: A theory of biotic acceptance.. Preslia.

[7] Vilà M, Pino J, Font X (2007). Regional assessment of plant invasions across different habitat types.. J Veg Sci.

[8] Chytrý M, Pyšek P, Tichý L, Knollová I, Danihelka J (2005). Invasions by alien plants in the Czech Republic: A quantitative assessment across habitats.. Preslia.

[9] Chytrý M, Maskell LC, Pino J, Pyšek P, Vilà M (2008b). Habitat invasions by alien plants: A quantitative comparison among Mediterranean, subcontinental and oceanic regions of Europe.. J Appl Ecol.

[10] Chytrý M, Wild J, Pyšek P, Tichý L, Danihelka J (2009). Maps of the level of invasion of the Czech Republic by alien plants.. Preslia.

[11] Pyšek P, Bacher S, Chytrý M, Jarošík V, Wild J (2010a). Contrasting patterns in the invasions of European terrestrial and freshwater habitats by alien plants, insects and vertebrates.. Glob Ecol Biogeogr.

[12] Pyšek P, Chytrý M, Jarošík V, Perrings C, Mooney HA, Williamson M (2010b). Habitats and land-use as determinants of plant invasions in the temperate zone of Europe.. Bioinvasions and globalization: Ecology, economics, management and policy.

[13] Chytrý M, Jarošík V, Pyšek P, Hájek O, Knollová I (2008a). Separating habitat invasibility by alien plants from the actual level of invasion.. Ecology.

[14] Pyšek P, Jarošík V, Kučera T (2002). Patterns of invasion in temperate nature reserves.. Biol Conserv.

[15] Usher MB (1988). Biological invasions of nature reserves: A search for generalisations.. Biol Conserv.

[16] Foxcroft LC, Rouget M, Richardson DM (2007). Risk assessment of riparian plant invasions into protected areas.. Conserv Biol.

[17] Gaston KJ, Jackson SF, Cantú-Salazar L, Cruz-Piñón G (2008). The ecological performance of protected areas.. Ann Rev Ecol Evol Syst.

[18] Vitousek PM, D'Antonio CM, Loope LL, Rejmánek M, Westbrooks R (1997). Introduced species: A significant component of human-caused global change.. New Zealand J Ecol.

[19] Lambdon PW, Pyšek P, Basnou C, Hejda M, Arianoutsou M (2008). Alien flora of Europe: Species diversity, temporal trends, geographical patterns and research needs.. Preslia.

[20] Hulme P, Pyšek P, Nentwig W, Vilà M (2009). Will threat of biological invasions unite the European Union?. Science.

[21] Margules CR, Sarkar S (2007). Systematic conservation planning.

[22] Richardson DM, Holmes PM, Esler KJ, Galatowitsch SM, Stromberg JC (2007). Riparian vegetation: Degradation, alien plant invasions, and restoration prospects.. Diversity Distrib.

[23] Pyšek P, Jarošík V, Kučera T (2003). Inclusion of native and alien species in temperate nature reserves: An historical study from Central Europe.. Conserv Biol.

[24] Alston KP, Richardson DM (2006). The roles of habitat features, disturbance, and distance from putative source populations in structuring alien plant invasions at the urban/wildland interface on the Cape Peninsula, South Africa.. Biol Conserv.

[25] Meek C, Richardson DM, Mucina L (2010). A river runs through it: Land use and the composition of vegetation along a riparian corridor in the Cape Floristic Region, South Africa.. Biol Conserv.

[26] Foxcroft LC, Jarošík V, Pyšek P, Richardson DM, Rouget M (2011). Protected-area boundaries as filters of plant invasions.. Conserv Biol.

[27] Cadenasso ML, Pickett STA, Weathers KC, Jones CG (2003). A framework for a theory of ecological boundaries.. BioSci.

[28] Richardson DM, Pyšek P, Rejmánek M, Barbour MG, Panetta FD (2000). Naturalization and invasion of alien plants: Concepts and definitions.. Diversity Distrib.

[29] Blackburn TM, Pyšek P, Bacher S, Carlton JT, Duncan RP (2011). A proposed unified framework for biological invasions.. Trends Ecol Evol.

[30] Cadotte MW, Murray BR, Lovett-Doust J (2006). Ecological patterns and biological invasions: Using regional species inventories in macroecology.. Biol Invas.

[31] Wilson JRU, Richardson DM, Rouget M, Procheş Ş, Amis MA (2007). Residence time and potential range: Crucial considerations in modelling plant invasions.. Diversity Distrib.

[32] Pyšek P, Jarošík V, Pergl J, Randall R, Chytrý M (2009). The global invasion success of Central European plants is related to distribution characteristics in their native range and species traits.. Diversity Distrib.

[33] Pyšek P, Jarošík V, Hulme PE, Kühn I, Wild J (2010c). Disentangling the role of environmental and human pressures on biological invasions across Europe.. Proc Nat Acad Sci U S A.

[34] Foxcroft LC, Rouget M, Richardson DM, MacFadyen S (2004). Reconstructing 50 years of *Opuntia stricta* invasion in the Kruger National Park, South Africa: Environmental determinants and propagule pressure.. Diversity Distrib.

[35] Wilson JRU, Gairifo C, Gibson MR, Arianoutsou M, Bakar BB (2011). Risk assessment, eradication, and biological control: Global efforts to limit Australian acacia invasions.. Diversity Distrib.

[36] Foxcroft LC, Richardson DM, Rouget M, MacFadyen S (2009). Patterns of alien plant distribution at multiple spatial scales in a large national park: Implications for ecology, management and monitoring.. Diversity Distrib.

[37] Foxcroft LC, Freitag-Ronaldson S (2007). Seven decades of institutional learning: Managing alien plant invasions in the Kruger National Park, South Africa.. Oryx.

[38] Foxcroft LC, Downey PO, Tokarska-Guzik B, Brock JH, Brundu G, Child L, Daehler CC, Pyšek P (2008). Protecting biodiversity by managing alien plants in national parks: Perspectives from South Africa and Australia.. Plant invasions: Human perception, ecological impacts and management.

[39] Koenig R (2009). Unleashing an army to repair alien-ravaged ecosystems.. Science.

[40] Foxcroft LC, Richardson DM, Wilson JRU (2008). Ornamental plants as invasive aliens: Problems and solutions in Kruger National Park, South Africa.. Environ Manag.

[41] Hui C, Foxcroft LC, Richardson DM, MacFadyen S (2011). Defining optimal sampling effort for large-scale monitoring of invasive alien plants: A Bayesian method for estimating abundance and distribution.. J Appl Ecol.

[42] MacFadyen S (2005). The Kruger National Park CyberTracker Program- Electronic Ranger Diaries.. http://www.sanparks.org/parks/kruger/conservation/scientific/gis/CyberTracker_Article.pdf.

[43] Kruger JM, MacFadyen S (2011). Science support within the South African National Parks adaptive management framework.. Koedoe.

[44] Gertenbach WPD (1983). Landscapes of the Kruger National Park.. Koedoe.

[45] van Wilgen BW, Nel JL, Rouget M (2007). Invasive alien plants and South African rivers: A proposed approach to the prioritization of control operations.. Freshwater Biol.

[46] Breiman L, Friedman JH, Olshen RA, Stone CG (1984). Classification and regression trees.

[47] Steinberg G, Colla P (1997). CART: Classification and Regression Trees.

[48] Steinberg D, Golovnya M (2006). CART 6.0 User's Manual.

[49] Breiman L (2001). Random Forests.. Machine Learning.

[50] Breiman L, Cutler A (2004). RandomForests™. An Implementation of Leo Breiman's RF™ by Salford Systems.

[51] Steinberg G, Colla (1995). CART: Tree-structured non-parametric data analysis.

[52] Cutler DR, Edwards TC, Beard KH, Cutler A, Hess KT (2007). Random forests for classification in ecology.. Ecology.

[53] Hochachka WM, Caruana R, Fink D, Munson A, Riedewald M (2007). Data-mining discovery of pattern and process in ecological systems.. J Wildlife Manag.

[54] Jarošík V, Simberloff D, Rejmánek M (2011). CART and related methods.. Encyclopedia of biological invasions.

[55] De'ath G, Fabricius KE (2000). Classification and regression trees: A powerful yet simple technique for ecological data analysis.. Ecology.

[56] Bourg NA, McShea WJ, Gill DE (2005). Putting a CART before the search: Successful habitat prediction for a rare forest herb.. Ecology.

[57] Hejda M, Pyšek P, Pergl J, Sádlo J, Chytrý M (2009). Invasion success of alien plants: Do habitat affinities in the native distribution range matter?. Glob Ecol Biogeogr.

[58] Hulme PE, Bacher S, Kenis M, Klotz S, Kühn I (2008). Grasping at the routes of biological invasions: A framework for integrating pathways into policy.. J Appl Ecol.

[59] Peterson AT, Papes M, Soberón J (2008). Rethinking receiver operating characteristic analysis applications in ecological niche modeling.. Ecol Modelling.

[60] Venette RC, Kriticos DJ, Magarey RD, Koch FH, Baker RHA (2010). Pest risk maps for invasive alien species: A roadmap for improvement.. BioSci.

