# Supplementary material for: Predicting Incursion of Plant Invaders into Kruger National Park, South Africa: The Interplay of General Drivers and Species-Specific Factors
Source: PLoS One. 2011 Dec 14;6(12):e28711. doi: 10.1371/journal.pone.0028711 (PMC3237482; doi:10.1371/journal.pone.0028711)
Supplement: Table S1 — Characteristics for the six focal species used in this study. (DOCX) [file pone.0028711.s001.docx]

**Table S1** Characteristics for the six focal species used in this study.

| **Species** | **Family** | **Region of origin** | **Life form** | **Reproduction** | **Dispersal vectors** | **Number of records in KNP^1^** | **Date first record in KNP** |
| --- | --- | --- | --- | --- | --- | --- | --- |
| *Ageratum houstonianum* Mill. | Asteraceae | Mexico, central America and the West Indies | Erect herb | Generative (achene) | Rivers, vehicles | 98 | 2000 |
| *Argemone ochroleuca* Sweet subsp. *ochroleuca* | Papaveraceae | Central America (Mexico) | Erect herb | Generative (achene) | Rivers, wind, contaminated soil | 228 | 1952 |
| *Chromolaena odorata* (L.) R. M. King & H. Rob. | Asteraceae | Central America and South America | Multi stemmed scrambling shrub | Generative (bristly achenes) | Rivers, wind, attaching with hooks to clothing, vehicles, road works  and farm machinery, seed contaminants | 327 | 1997 |
| *Lantana camara* L. | Verbenaceae | Central America and South America | Compact, floriferous shrub or untidy scrambler | Generative (fleshy drupes) | Rivers, frugivorous birds | 2332 | 1940 |
| *Opuntia stricta* (Haworth.) Haworth. | Cactaceae | North America and West Indies (Florida, Texas, Cuba) | Multi-branched succulent shrub | Generative (succulent berries) and vegetative | Small mammals (mice, but not observed in KNP), baboons, monkeys, elephant, frugivorous birds (but not observed in KNP) | 1663 | 1953 |
| *Xanthium strumarium* L. | Asteraceae | Uncertain, probably Central and South America | Much-branched erect herb | Generative (one seeded achene) | Animals, humans, rivers | 274 | 1953 |

Sources:

Global Invasive Species Database (2011) Available from

<http://www.issg.org/database/welcome> (Accessed 02/08/2011)

Henderson L (2001) Alien weeds and invasive plants. Pretoria: Plant Protection Research

Institute, Agricultural Research Council.

Pacific Island Ecosystems at Risk (2011) Available from [http://www.hear.org/pier](http://www.hear.org/pier/) (Accessed

02/08/2011)

Wells MJ, Balsinhas H, Joffe H, Engelbrecht VM, Harding G, Stirton CH (1986) A catalogue

of problem plants in southern Africa. Pretoria: National Botanical Institute.

^1^ Records up to and including 2007
